# Design and analysis of umbrella trials: Where do we stand?

**DOI:** 10.3389/fmed.2022.1037439

**Published:** 2022-10-12

**Authors:** Luke O. Ouma, James M. S. Wason, Haiyan Zheng, Nina Wilson, Michael Grayling

**Affiliations:** ^1^Biostatistics Research Group, Population Health Sciences Institute, Faculty of Medical Sciences, Newcastle University, Newcastle upon Tyne, United Kingdom; ^2^Medical Research Council (MRC) Biostatistics Unit, University of Cambridge, Cambridge, United Kingdom

**Keywords:** stratified medicine, master protocol, precision medicine, precision oncology, adaptive design, biomarker-guided design, innovative design

## Abstract

**Background:**

The efficiencies that master protocol designs can bring to modern drug development have seen their increased utilization in oncology. Growing interest has also resulted in their consideration in non-oncology settings. Umbrella trials are one class of master protocol design that evaluates multiple targeted therapies in a single disease setting. Despite the existence of several reviews of master protocols, the statistical considerations of umbrella trials have received more limited attention.

**Methods:**

We conduct a systematic review of the literature on umbrella trials, examining both the statistical methods that are available for their design and analysis, and also their use in practice. We pay particular attention to considerations for umbrella designs applied outside of oncology.

**Findings:**

We identified 38 umbrella trials. To date, most umbrella trials have been conducted in early phase settings (73.7%, 28/38) and in oncology (92.1%, 35/38). The quality of statistical information available about conducted umbrella trials to date is poor; for example, it was impossible to ascertain how sample size was determined in the majority of trials (55.3%, 21/38). The literature on statistical methods for umbrella trials is currently sparse.

**Conclusions:**

Umbrella trials have potentially great utility to expedite drug development, including outside of oncology. However, to enable lessons to be effectively learned from early use of such designs, there is a need for higher-quality reporting of umbrella trials. Furthermore, if the potential of umbrella trials is to be realized, further methodological research is required.

## 1. Introduction

The advent of precision medicine is progressively revolutionizing the conduct of clinical trials. The high cost and vast resources required to conduct clinical trials implies that it would be slow and inefficient to test targeted therapies in a large number of small patient sub-populations under the conventional paradigm of a single treatment vs. standard of care. Increasingly, a common protocol (called a “master protocol”) is being utilized to test multiple targeted treatments within a single disease setting in different subtrials. These subtrials are defined by disease subtype or patient-level characteristics that are thought to be associated with treatment response. Such a master protocol is commonly referred to as an *umbrella trial* (1, 2).

Conceptually, an umbrella design is simply a set of (sub)trials run in parallel. It offers a selection of appealing advantages, including: (i) that several treatment-related questions can be answered in a single trial, (ii) a potential reduction in the number of patients required (for instance, by including a common control arm), and (iii) expedited drug development, with shorter trial duration and lower costs overall, relative to running traditional clinical trials independently (3, 4). However, numerous statistical complexities may arise in the conduct of umbrella trials, including but not limited to a desire for adaptive design elements, the choice between Bayesian/frequentist decision rules, appropriate sample size calculation, whether to borrow information, and how to control particular error rates. The solution that accommodates such considerations will typically vary depending on the variant of umbrella design chosen and study-specific requirements; for instance, conducting a late-phase umbrella trial would mean more stringent requirement on error-rate control. More recently, complex (hybrid) designs have also emerged that blur the lines between umbrella designs and other related master protocol designs, which present even more interesting statistical questions.

Reviews from 2018 to 2019 found that nearly all umbrella trials have been implemented to date in oncology, with the majority being either phase I or II, and incorporating the use of randomization (5, 6). Notably, there are fewer implemented umbrella trials relative to other key master protocols (6, 7): platform trials (which allows flexible addition of new treatment arms or patient subgroups) and basket trials (where a targeted therapy is evaluated across multiple diseases having a common therapeutic target).

The relative rarity of umbrella trials may speak in part to the fact that despite their noted advantages, there remains limited guidance on their design and analysis. By contrast, several designs have been proposed for basket and platform trials; these vary by the utilized statistical analysis techniques, the decision rules that can be incorporated, and cover different purposes or phases of drug development (8–16). Whilst there have been a number of recent reviews of master protocols, they have focused on delivering landscape analyses of master protocols in general, providing often high-level discussions of various trial designs, their definitions in the literature, and key published examples (3, 5, 6, 17). Though several works have discussed statistical analysis methods for master protocols, they have focused on basket and platform trials (7, 15). Moreover, as we argue further later, the current and future potential of umbrella trials in non-oncology settings is enormous (18, 19). So far, though, published articles on umbrella trials have almost exclusively discussed oncology related considerations.

In this article, we conduct a comprehensive review with a focus on (i) the design and analysis of recent umbrella trials, and (ii) proposed trial designs and statistical methods available for umbrella trials. Our objectives are two-fold: (1) to provide awareness of the statistical considerations for the design and analysis of umbrella trials, with attention paid to non-oncology settings, and (2) to highlight areas in which further research is required. In this way, we hope to lay clear the state of play for umbrella designs.

The remainder of this article is structured as follows. We proceed by describing our approach to identifying relevant literature in Section 2. We then lay out the general umbrella design framework in Section 3, before describing characteristics of conducted umbrella trials in Section 4. Analysis and design strategies for umbrella trials are discussed in Sections 6 and 5, respectively, before links to related designs are drawn in Section 7. In Section 8, we highlight some open questions for umbrella trials and close with a discussion of our findings in context in Section 9.

## 2. Review methodology

Key elements of our approach to identifying published articles relating to umbrella trials are listed here. Further details including PRISMA checklists (20) are provided in the Supplementary materials.

### 2.1. Data sources and search strategy

We conducted a systematic search of literature present in PubMed, checking articles present by 12th May 2021 for relevance to umbrella trials. To overcome the common mislabeling/misclassification of master protocol designs (21), we extracted articles that stated they were related to umbrella, basket, or platform trials. Our exact search term is provided in the Supplementary material. We complemented our search with a review of published master protocol reviews and manual forward/backward citation checks of relevant articles. This review was not registered.

### 2.2. Article inclusion criteria

The literature search aimed to identify articles that either (i) proposed statistical methodology for umbrella trials, (ii) reported the protocol/results of an umbrella trial, or (iii) discussed other considerations related to umbrella trials. Inclusion criteria (iii) was flexible to accommodate the variable definitions of master protocols in the literature; several identified master protocols did not neatly fit the widely accepted definition of an umbrella trial (1), but were closely related and provided useful information for umbrella trials. For example trials such as NCI-MATCH, CUSTOM have been labeled as umbrella trials by some authors (3, 21), others consider them as a basket trial (1, 21), while others (3, 7) agree they fit neither classification. Strictly, NCI-MATCH and CUSTOM are not umbrella trials but hybrid designs.

All abstracts were independently reviewed by two of the five authors to check for whether they met one or more of the three inclusion criteria. Conflicts regarding study relevance were then resolved by a third reviewer to create a final list of relevant articles.

### 2.3. Data extraction and synthesis

For relevant articles that presented a clinical trial protocol/results, data were extracted independently by two of the five authors using a standardized data extraction form (see Supplementary material). Specifically, we extracted information on trial phase, disease area, the number of subtrials/modules, the sample size, the approach to sample size calculation, randomization, error rate control, the primary outcome, any adaptive features, the analysis approach, and whether patients were eligible for multiple subtrials, amongst other considerations. The independently extracted data were then synthesized by MG into a single dataset, from which the descriptive overview of the characteristics of these trials is provided. The details of other relevant articles (e.g., those providing statistical methodology) is described narratively in relevant sections of this article.

## 3. The umbrella trial framework

We now proceed by describing umbrella trials in one general framework. An umbrella trial recruits patients with a single condition (e.g., Alzheimer's disease); patients with this condition are screened for whether they belong to one of *S* pre-defined subgroups, also called modules. These modules or subgroups comprise the different subtrials. We will use *s* = 1, …, *S* to indicate the subgroup to which a patient belongs. In cancer trials, subgroups are typically defined by patients exhibiting a particular genetic marker, but we will discuss examples later of more general definitions from areas outside of oncology. In some cases, an umbrella trial may also adopt an “all comers” type approach, in which patients with the condition of interest who are screened but found to not belong to any of the *S* subgroups may be offered the opportunity to enter a separate subtrial. For brevity, we omit this consideration from most of our discussions that follow, but note that when such a separate subtrial is included, little is lost by simply considering this as a separate subgroup with its own index.

Umbrella trials can then be defined by a rule that links the subgroup a patient belongs to with the possible allocation to treatments. We assume that all possible treatment assignments can be split such that there are *T*_*C*_≥0 and *T*_*E*_≥1 treatments that are considered to be control and experimental assignments, respectively. We use *t*_*C*_ = 0, …, *T*_*C*_ and *t*_*E*_ = 1, …, *T*_*E*_ to index these treatments. Then, an umbrella trial functions such that patients in subgroup *s* are allocated, subject to a chosen randomization method, to one of the treatments in set C_*s*_∪E_*s*_, where C_*s*_ and E_*s*_ are subsets of the *T*_*C*_ control and *T*_*E*_ experimental arms.

**Figure 1:**
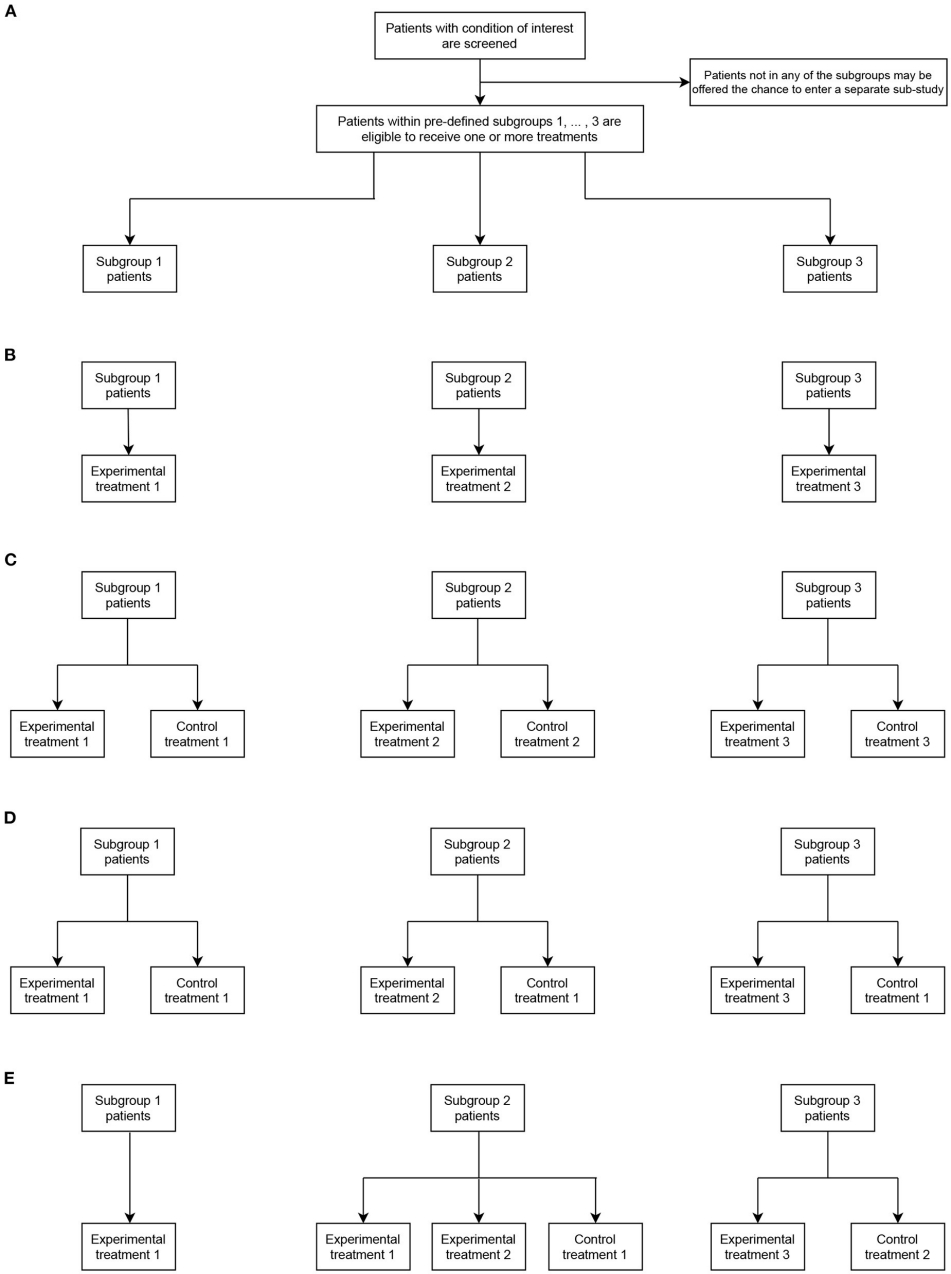
Representative umbrella trial designs in the case of three subgroups (*S* = 3) and three experimental treatment arms (*T*_*E*_ = 3). **(A)** indicates the fundamental structure of a trial of this type. **(B–E)** give different design options allowing assignment to the experimental and possibly control treatments in each subgroup (as defined by the values of *T*_*C*_, C_*s*_, and E_*s*_).

To illustrate this more clearly, Figure 1 shows four example umbrella designs in the case that *S* = 3 and *T*_*E*_ = 3, for possible values of *T*_*C*_ and possible choices of the sets C_*s*_ and E_*s*_. Figure 1A indicates the principal structure of the trial and then Figures 1B–E correspond to the four types, respectively. These are

- Figure 1B: A non-randomized umbrella trial, in which *T*_*C*_ = 0 (and thus C_*s*_ = ∅ for *s* = 1, 2, 3) and E_*s*_ = *s* for *s* = 1, 2, 3. That is, patients in subgroup *s* are automatically assigned to a linked experimental treatment.
- Figure 1C: A randomized umbrella trial, in which *T*_*C*_ = 3, with C_*s*_ = E_*s*_ = *s* for *s* = 1, 2, 3. That is, patients in subgroups *s* are assigned to either a linked experimental treatment, or a linked control treatment.
- Figure 1D: A randomized umbrella trial, in which *T*_*C*_ = 1, with C_*s*_ = 1 and E_*s*_ = *s* for *s* = 1, 2, 3. That is, unlike in Figure 1B, patients in subgroup *s* are assigned to either a linked experimental treatment, or a common control treatment.
- Figure 1E: An example of a “mixed” form of umbrella trial, in which *T*_*C*_ = 2. Here, all patients in subgroup *s* = 1 are assigned to experimental treatment 1 (i.e., non-randomized allocation with C_1_ = ∅ and E_1_ = 1). Whereas, in subgroup *s* = 2, patients can be assigned to one of two possible experimental treatments or a control arm (i.e., randomized allocation with C_2_ = 1 and E_2_ = {1, 2}). Similarly, in subgroup *s* = 3, patients can be assigned to one of two possible experimental treatments or a control arm (i.e., randomized allocation with C_3_ = 3 and E_2_ = {2}).

We return later to consider examples of several of these possible umbrella designs that have now been used in practice.

## 4. Characteristics of conducted umbrella trials

**Figure 2:**
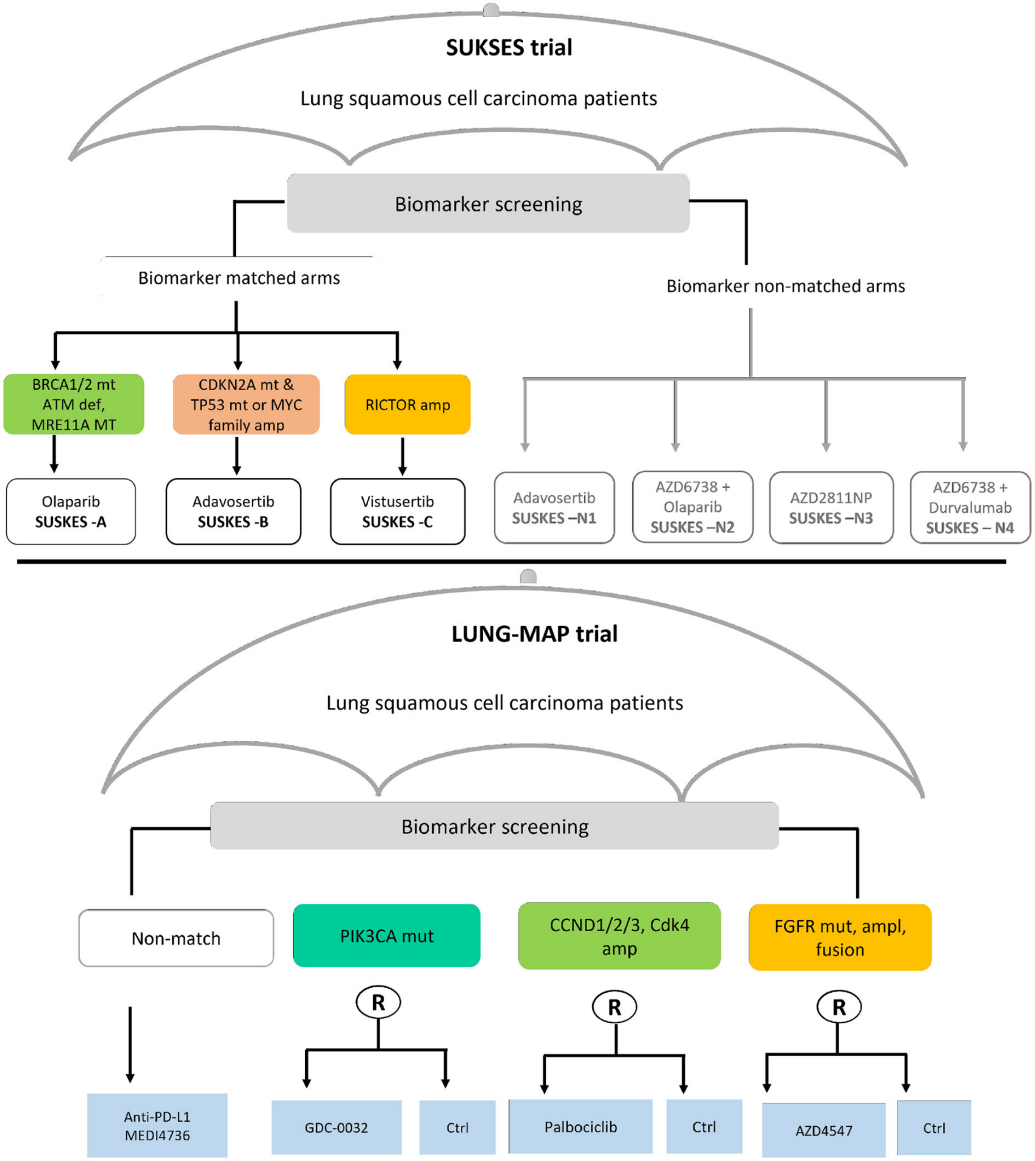
Two examples of real umbrella trials featuring distinct designs. The schema in the top panel depicts the small cell lung cancer umbrella korea studies (SUKSES–NCT02688894) trial. The bottom panel demonstrates the study schema of the lung cancer master protocol (Lung-MAP–NCT03851445) trial.

In this section, we present findings of the review on statistical aspects of the conducted umbrella trials. A total of 1,789 articles were identified in the updated search; 1,652 articles were excluded in the first screening by title and abstract; and 137 articles were assessed for full text eligibility. Seventy-seven articles were included in the final data extraction with 42 of these identified from forward/backward citation checks of articles naming umbrella trials. Supplementary Figure 1 of the Supplementary material depicts the search and selection procedure using the PRISMA (20) flow-diagram. Thirty-eight umbrella trials, all investigating experimental drugs, were identified in our review; a summary of their key characteristics is presented in Tables 1, 2. The relevant umbrella trials featured different designs; see Figure 2 which illustrates two ongoing umbrella trials in lung cancer, the Lung-MAP (22) and SUSKES (23) trials.

**Table 1:** Overview of the characteristics of the 38 identified umbrella trials.

**Category**	***n* (%)**
Trial phase
Early phase (I, II)	23 (60.5)
Late phase (III-IV)	3 (7.9)
Seamless (I/II, II/III, III/IV)	10 (26.3)
Unclear	2 (5.3)
Disease setting
Oncology	35 (92.1)
Non-oncology	3 (7.9)
Primary endpoint
Binary & TTE^a^	2 (5.3)
Safety & Binary^a^	1 (2.6)
Safety & TTE^a^	2 (5.3)
TTE^b^	9 (23.7)
Safety	1 (2.6)
Binary	18 (47.4)
Continuous	2 (5.3)
Unclear	3 (7.9)
Primary endpoint same across subtrials
Yes	27 (71.1)
No	7 (18.4)
Unclear	4 (10.5)
Treatment allocation
Randomized	12 (31.6)
Non-randomized	14 (36.8)
Both (randomized and non-randomized)	7 (18.4)
Unclear	5 (13.2)
Control same across subtrials^c^
Yes	4/12 (33.3)
No	4/12 (33.3)
Unclear	4/12 (33.3)
Overlapping/Mutually exclusive subgroups
Overlapping	8 (21.1)
Mutually exclusive^d^	22 (57.9)
Unclear	8 (21.1)
Approach to deal with overlapping subgroups
Yes	7/8 (87.5)
No	1/8 (12.5)

The denominator for the percentages is 38 unless otherwise stated. The term Unclear means that there was insufficient information available in identified data sources for classification. ^a^Co-primary endpoints; ^b^TTE is a time-to-event endpoint such as overall survival or progression free survival; Binary endpoints—overall/partial response, disease control rate; Safety endpoints—adverse events, dose limiting toxicity, maximum tolerated dose; ^c^Assessed only for randomized trials; ^d^Subtrials/subgroups are mutually exclusive if no patients have tested positive for >1 subgroup-treatment defining criteria (i.e., biomarkers).

**Table 2:** Statistical considerations in the design and analysis of the 38 identified umbrella trials.

**Statistical consideration**	***n* (%)**
Adaptive features
Yes	15 (39.5)
No	5 (13.2)
Unclear	18 (47.4)
Sample size calculation
Pooled	4 (10.5)
Separate	13 (34.2)
Unclear	21 (55.3)
Primary analysis framework
Frequentist	15 (39.5)
Bayesian	2 (5.3)
Bayesian and Frequentist	1 (2.6)
Unclear	20 (52.6)
Indicated an error rate had been controlled
Yes	13 (34.2)
No	1 (2.6)
Unclear	24 (63.2)
Error rate control consistent with design^a^
Yes	7/13 (53.8)
No/Unclear	6/13 (46.2)
Target effect size same across subgroups
Yes	4 (10.5)
No	11 (28.9)
Unclear	23 (60.5)
Described how missing data would be handled
Yes	0 (0.0)
No	38 (100.0)

The denominator for the percentages is 38 unless otherwise stated. The term Unclear means that there was insufficient information available in identified data sources for classification. ^a^Assessed only for relevant trials that reported error rate control in the design.

Most umbrella trials were conducted in oncology (92.1%, 35/38), mainly in non-small cell lung cancer, followed by breast cancer. Only three trials were conducted in non-oncology settings, namely, HIV (ACTG A5288) (24), Alzheimer's disease (DIAN-TU) (25), and rheumatoid arthritis (TOZURA) (26, 27). Unlike the oncology trials that evaluated drugs that targeted well-validated oncogenes now used in many trials, the non-oncology trials evaluated types of biomarkers. For instance, the DIAN-TU trial tested a diverse panel of imaging and fluid Alzheimer's biomarkers while resistance to nucleoside reverse transcriptase inhibitors was the major determinant of treatment assignment in ACT A5288.

More than two-thirds (73.7%, 28/38) were conducted in an early phase setting (phase I or II), and ten (26.3%) trials had a seamless design (5 phase I/II, 4 phase II/III, 1 phase III/IV). Fifty-percent of the trials incorporated randomization in some way (19/38); 12 studies (31.6%) randomized patients in all subtrials, while 7 studies (18.4%) randomized patients in specific subtrials only. Four of the twelve (33.3%) completely randomized studies used a common control treatment across the subtrials, with four trials (33%) also using a different control in at least one subtrial.

The median number of subtrials was 5 (IQR: 3–9) amongst the 30/38 trials (78.9%) that reported the number of subtrials. In most trials (55.3%, 21/38), all of the subtrials were mutually exclusive, but in eight trials (21.1%) there were overlapping treatment defining characteristics (such as biomarkers). Only seven trials clearly reported whether patients were eligible for multiple subgroups alongside an approach for allocating such patients to a suited treatment. In these seven trials, the approaches used for allocating patients eligible for multiple subgroups to treatments included a hierarchy of biomarkers approach (28, 29), weighting by the prevalence of subgroups (30), a ranking algorithm (31), and a prioritization approach incorporating both allele frequency and gene expression (32).

The median planned overall sample size of the umbrella trials was 259 participants (IQR: 135-4936). However, we note that roughly one-third of the studies did not report the overall or per subtrial sample sizes (26.3%, 10/38). The sample size per “module” was highly varied across trials; for instance in FUTURE (33), each of the 7 modules had 20 patients, the module sample sizes in UPSTREAM (34) were 55, 55, 55, 32, 32, 20, 40, and 76, respectively, while the module sample sizes in MORPHEUS (35) was 435, 410, 290, 382, 126, 280.

More than half of the umbrella studies used the same primary outcome across the subtrials (52.6%, 20/38); binary outcomes were the most common (71.1%, 27/38; overall response rate was used in 17 studies) followed by time-to-event outcomes (28.9%, 11/38). Eight trials (21.1%) had co-primary outcome measures; for example PFS and ORR (34, 36) and PFS and OS (22, 28, 37).

Nearly two-fifths (39.5%, 15/38) of the trials enabled mid-course adaptations to the design. This was in spite of the fact that in nearly half of the trials (47.4%, 18/38) there was insufficient information to deduce whether or not any adaptive features had been included. The most commonly allowed adaptation was futility/efficacy monitoring (60.0%, 9/15). Other adaptive features observed were adaptive randomization (20.0%, 3/15) (38, 39), sample size re-estimation (6.7%, 1/15) (40), addition of new arms (13.3%, 2/15) (23, 41), and a two-stage design to terminate ineffective treatments in stage I (20%, 3/15) (23, 42, 43).

We observed considerable differences in the information available about umbrella trials conducted to date. In at least half of the umbrella trials identified it was impossible to clearly ascertain information about the approach to sample size calculation (55.3%, 21/38), the error rates controlled (63.2%, 24/38), the statistical framework (to be) used in the analysis (Bayesian or frequentist) (52.6%, 20/38), whether pooling or borrowing of information across subtrials was permitted (50.0%, 19/38), and any considerations for dealing with missing data (100.0%, 38/38). We return to discuss this point again in the Discussion.

## 5. Design and sample size calculation

With many variants of umbrella trial possible, numerous statistical considerations for their design and analysis can arise. In this section, we discuss several key considerations for good practice.

### 5.1. Adaptive vs. non-adaptive design

**Figure 3:**
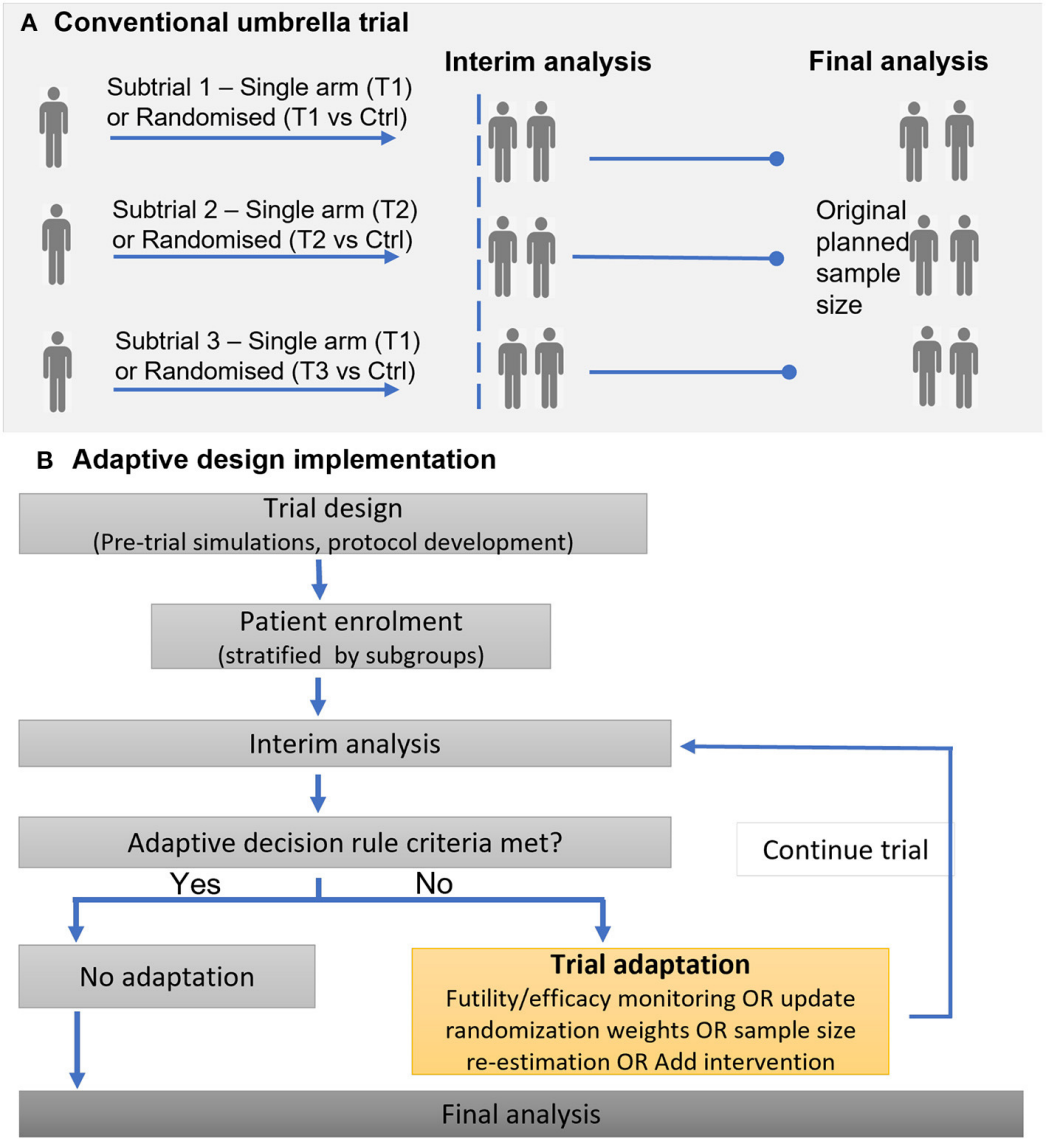
Illustration of implementing a conventional (non-adaptive) umbrella trial and an adaptive umbrella trial. **(A)** Conventional umbrella trial. **(B)** Adaptive design implementation.

Adaptive designs provide the opportunity to undertake mid-trial changes, such as adding or dropping of arms, or adjustment in treatment allocation ratios and sample size based on accrued data. Such trial adaptations are desirable to improve overall trial efficiency, for instance enrolling fewer patients but maintaining the desired statistical power, or allocating a higher proportion of patients to the best treatment option (44). Figure 3 illustrates a general framework for the implementation of an adaptive umbrella design involving biomarker subgroups.

Adaptive features such as response-adaptive randomization, addition of arms, or stopping for futility/efficacy, are common in recent and ongoing umbrella trials; in all, 15 studies (39.5%) incorporated adaptive features into their design. The Lung-MAP trial is a classic example that has undergone adaptations (22, 45, 46): (i) new subtrials have been initiated (ii) the trial was modified from a phase II/III trial to include both phase II and phase III evaluations, leading to the inclusion of single-arm studies and modification of the “non-match” subtrial from a randomized to single-arm design.

Several proposed statistical methods for the design and analysis of umbrella trials have incorporated one or more adaptive features (47–49), though it does not appear any have yet been implemented in practice. Wason et al. (47) proposed a Bayesian adaptive design for use in biomarker-guided umbrella trials. In this design, patients are initially equally randomized between treatments they are eligible for. Then, at interim analyses accruing trial data are used to update the allocation probabilities; this is done by computing, for each biomarker profile, the posterior probabilities that an experimental treatment is superior to control. The design of Wason et al. (47) works well when pre-specified biomarker-treatment pairings are correct and in scenarios where unintended biomarker-treatment pairings are observed. Unintended biomarker-treatment pairings are instances when a treatment gives benefit to a biomarker sub-population for which it is not primarily targeted in a biomarker-guided trial.

The aforementioned HCOMBS methodology proposed by Kang et al. (49) uses interim futility clustering in a two-stage phase II umbrella trial, allowing for early dropping of inactive arms. At the interim analysis, treatment arms are grouped into active or inactive subgroups based on whether the response rates are less than or greater than pre-specified thresholds. Treatment arms in the inactive subgroups are dropped for futility while arms in the active group advance to the next stage.

Ballarini et al. (48) proposed a two-stage Bayes optimal umbrella trial in two disjoint disease subgroups. In the first stage, the proportion of patients to be recruited from each biomarker subgroup is optimized, along with the timing of the interim analysis. In the second stage, they update the optimal allocation based on results at the interim analysis. Ren et al. (50) also proposed a statistical framework for a three-arm phase III umbrella trial with a common control allowing addition of a treatment arm mid way through the trial.

The potential advantages of adaptive designs notwithstanding, we end this subsection by highlighting that although adaptive features can provide several efficiency gains to a trial, trialists must weigh their pros and cons as they do not always provide an advantage. For example, when the clinical outcome of interest is a long-term endpoint, adaptive designs are not recommended (44).

### 5.2. Patient eligibility for multiple subgroups

As discussed in Section 4, patients with multiple actionable biomarkers are seen in several umbrella trials (3, 4). This raises the question of how to address in the design such patients who test positive for multiple biomarkers (i.e., patients are often eligible for multiple subgroups). Sometimes, it is not clear which biomarker is a potential disease driver for such a patient (51). The allocation of these patients to treatment is not straightforward; the decision may either be left to the trial clinician(s), or different simple and complex approaches can be used. In this review, for more than half of the umbrella trials identified (21/38, 55.3%) it was not clear whether patients were eligible for multiple subgroups. Furthermore, even when there was clear evidence of eligibility to multiple subgroups, as in National Lung Matrix (36, 52) and MODUL (42), reporting on how these patients would be dealt with was typically unclear. In all, it was clear that for seven trials patients were eligible for multiple treatment subgroups, but only five clearly reported the approach to deal with the allocation of such patients: weighted allocation based on prevalence of subgroups [Lung-MAP (22)], a hierarchy of biomarkers approach [FOCUS4 (28)], a ranking algorithm [N2M2 (31)], a pre-specified prioritization approach [PANGEA (32)], and assignment to non-matched arms [SUKSES (23)].

Ouma et al. (53) and Kesselmeier et al. (54) have demonstrated that when overlapping subgroups are likely, an appropriate treatment allocation strategy should be pre-specified, to ensure desired trial operating characteristics are maintained. Kesselmeier et al. (54) compared two approaches: (i) pragmatic allocation to the eligible subtrial with fewer patients and (ii) equal randomization. They showed that as the proportion of patients who test positive for multiple biomarkers increases, the estimated treatment effects will increasingly deviate from those obtained from the corresponding independent trials. That is, if the issue of multiple biomarkers is not addressed appropriately in the design and analysis, biased treatment effects are very likely. Ouma et al. (53) investigated the performance of various approaches previously used in practice and other plausible strategies, namely, the hierarchy approach, Bayesian adaptive randomization (BAR), equal randomization, constrained randomization, and randomization with a fixed allocation probability to control. They report the impact of treatment allocation strategies on operating characteristics such as statistical power, treatment allocation ratios, proportion of patients on the best treatment available to them, and accuracy and precision of treatment effect estimates. Although Ouma et al. (53) showed that no treatment allocation strategy is optimal in all settings, the hierarchy and BAR approaches performed best in general. However, the hierarchy approach is heavily dependent on the validity of the pre-specified hierarchy of subgroups and performs poorest if the hierarchy is wrong. A notable advantage of BAR is that it favors the allocation to treatments that are likely to be more beneficial.

One practical consideration is that when the proportion of patients with multiple biomarkers is small, an approach such as equal randomization is sufficient. In contrast, in scenarios where the prevalence of overlapping subgroups is unknown, one may argue for the use of accruing trial data to adaptively inform the treatment allocation decisions.

### 5.3. Adjustments for testing multiple null hypotheses

Error rate control is often strictly required for confirmatory trials; our review's findings affirm the general consensus that error rate control is less stringent in early phase trials. Thirteen studies reported on control of error rates, one study (24) had no error rate control, while for 24 studies we could not establish whether error rates were controlled. Of the 13 studies which stated error rates controlled, type I and type II error rate control for each hypothesis was the most common approach (12/13, 92.3%), while one trial (32) reported computation of pooled type-I and type-II errors. The family-wise error rate was controlled in two trials, WSG ADAPT (55) and BATTLE-2 (39). Notably, we were only able to establish that error rate control in the analysis was consistent with the design for seven studies.

In the standard (most commonly implemented) umbrella design (Figure 1B), the hypotheses being tested relate to different experimental treatments investigated in different disease subgroups and therefore multiplicity adjustment is not required (48, 50, 56–58). However, if adaptive design features are used, it is important to ensure error rates are controlled across stages, at least per subtrial.

In Figure 1E where some experimental treatments are evaluated in multiple subgroups, multiplicity correction is usually needed in confirmatory trials. Collignon et al. (58) present some further considerations for error rate control in umbrella trials. In settings where patients test positive for multiple biomarkers (say, B1 and B2) and there is no rationale for preferential enrolment to a specific sub-study, investigators may decide to randomize patients between the biomarker-defined sub-studies instead of an elective approach. Within each sub-study, they can be further randomized to experimental treatments. A potential concern is when multiple biomarker patients (B1+B2+) are randomized to the sub-studies while B1+ and B2+ are assigned to respective sub-studies B1 and B2. This may lead to over-representation of the multiple biomarker patients in one sub-study and consequently the need to control possible inflation of the “master protocol–wise error rate” (MPWER). The MPWER is the “the probability of declaring at least one of the subtrials of the master protocol positive when none is” (58). Another situation where maintaining error rates is important is when information borrowing is incorporated between certain common treatment arms, for example the control arm in Figure 1D. This requires careful evaluation by pre-trial simulations if the potential implications for error rates is to be well-understood.

Besides the aforementioned considerations for error rate control, there are some recent methodological developments on this subject relevant to umbrella trials. Werner et al. (59) proposed *population-wise error rate* (PWER) for multiple type-I error control when a treatment is tested in two subgroups that are not disjoint. This is specific to Figure 1E, if subgroup 1 and 2 are not overlapping, yet independent conclusions are desired for each disease subgroup. The underlying principle for this proposal is that the intersections for S1 and S2 will contain patients that are possibly exposed to multiple erroneously rejected null hypotheses, implying that one has to adjust for multiplicity. Werner and colleagues have shown that PWER is less conservative and is associated with increased power compared to the FWER (59). To the best of our knowledge, PWER has not been implemented in a software or real trials. We also point the reader to relevant statistical methodologies for Bayesian and/or adaptive umbrella trials by Kang et al. (49), Ren et al. (50), and Ballarini et al. (48) discussed in various sections in this manuscript that have shown promising control of error rates.

### 5.4. Sample size computation

The sample size determination criteria was unclear for most umbrella trials in our review (21/38, 55.3%). Amongst trials that reported sample size computation, 76.5% (13/17) determined the number of patients required for each subtrial separately. Besides, we identified a single methodology that proposed a novel sample size determination criteria. Kang et al. (49) in their Bayesian hierarchical design used a grid-search algorithm to determine the optimal sample size per subtrial that attained a desired marginal power and FWER. Their proposed methodology enabled borrowing of information and required fewer patients than independent Simon's design for each subtrial.

One argument for the separate approach is the visualization of an umbrella trial as a collection of independent trials conducted in parallel, each testing a unique hypothesis that is unrelated to those tested in other subtrials. For example, the Lung-Map and ALCHEMIST subtrials are designed independently based on biomarker prevalences, whilst maintaining the same design parameters across all subtrials (22, 30). Another reason to use the separate approach is when certain key design parameters, for instance the target effect size or choice of primary outcome, vary across subtrials: nine studies in our review had different effect sizes across subtrials, while seven had at least one subtrial using a different endpoint to the rest.

The idea of pooled sample size computation for umbrella trials is relatively uncommon in practice; only four trials (ACTG A5288, BATTLE-1&2, PANGEA), used a pooled approach (60–62). Since a generic approach to sample size computation that works for different umbrella designs is currently lacking from the literature, the use of simulation for umbrella trial design is often required. This will especially be true if sample size calculation is to be carried out accounting for a Bayesian analysis that facilitates borrowing of information. Little work has been conducted to date, though, to help guide sample size calculation in this manner for umbrella trials.

We highlight that, in practice, some biomarkers of interest may have rather low prevalence. It could therefore be unrealistic to power the corresponding subtrials in isolation, unless targeting a large effect size with a higher type I error and lower power than would otherwise be common (3).

### 5.5. Design of umbrella trials by phase of development

We identified umbrella trials spanning the full spectrum of drug development (phase I–IV). However, most trials in our review were early phase trials. All of the identified phase II/III or phase III designs incorporated randomization into their design. Error rate control was less common in early phase trials; only 1/7 (14.3%) phase I or phase I/II trials and 8/20 (40%) phase II trials mentioned the control of type I and type II errors. Late phase trials were, as would be expected, generally larger in sample size; in our review the median sample size for phase I, II, and III trials was 154, 229, and 770 participants, respectively.

Statistical considerations for umbrella trials vary depending on the phase of application. In early phase trials, smaller sample sizes are usually targeted for feasibility reasons. In phase I, the umbrella framework in general offers logistical rather than statistical advantages, with the subtrials typically comprising a single-arm (preventing, e.g., the potential for borrowing of information). Larger phase II trials (e.g., phase IIB) offer greater potential for added efficiencies in an umbrella context, as there is generally larger scope for the use of novel methodologies. In the confirmatory setting, the standard two-arm randomized-controlled trial framework implies that there may be less scope for statistical efficiencies (such as borrowing information across subgroups) to be achieved from an umbrella design, although logistical advantages may still be realized. In particular, stringent control of error rates is often required in phase III to meet regulatory requirements, which may negate the possibility to borrow information (63, 64). However, some authors have proposed confirmatory umbrella designs with high power and desirable type I error rate control (48, 50).

## 6. Analyzing umbrella trial data

The appropriate analysis strategy for an umbrella trial depends on its design and objectives. In particular, both Bayesian and frequentist analysis frameworks have utility in the analysis of umbrella trial data, and within each of these paradigms there have been a few established approaches that may be employed. In the following, we summarize key considerations in this regard, in particular around the potential for borrowing of information using examples identified in our review.

### 6.1. Bayesian vs. frequentist approaches: General considerations

To date, it is evident a frequentist framework is the dominant approach for umbrella trial analysis; in our review, nearly all trials where the statistical framework used in the analysis was reported (18/38, 47.4%) used a frequentist framework (16/18, 88.8%). Most commonly, this involves analyzing the subtrials separately (also called stand-alone analysis).

Bayesian methods are gaining popularity more generally for master protocols (15, 65), especially Bayesian adaptive approaches to enable efficient pooling of results when subtrials have common features such as similar treatment(s) or control(s) (e.g., the randomized umbrella trial in Figure 1D). Indeed, we identified two umbrella trials that were analyzed under the Bayesian framework, as well as one trial that used both frequentist and Bayesian approaches.

Much of the literature on Bayesian methodologies for umbrella trials has only leveraged Bayesian techniques to optimize certain features of the trial; the primary analysis has still employed frequentist hypothesis testing (47–50). This may be argued to be advantageous from the perspective of error-rate control. The Bayesian approach has the ability to allow incorporation of information obtained within or outside of the trial into the analysis. For example, based on pre-trial evidence, one may incorporate prior beliefs about the efficacy of targeted treatments by specifying informative priors for treatment-subgroup interactions (47). Additionally, historical control (or experimental treatment) information from different sources can be formulated into robust priors to inform certain aspects of trial design, such as sample size calculation (66). However, the use of historical information has not been extensively explored in the umbrella trial setting, although the advantages and potential limitations have been documented (67). Given the complexity associated with the design of many umbrella trials, the often stated advantages of Bayesian inference in their ease of interpretation for non-statisticians, may also be found to have profound advantages.

Both Bayesian and frequentist frameworks can also be used in the statistical analysis of umbrella trials. As noted above, this may be in the form of using Bayesian decision rules at interim analyses to guide mid-trial adaptations while retaining a frequentist final analysis. Alternatively, both Bayesian and frequentist analyses could be performed, with the frameworks adopted for different endpoints (e.g., computing the Bayesian posterior probability for a binary outcome alongside a frequentist Cox regression for a survival endpoint). An example of using both Bayesian and frequentist approaches is the BATTLE-1 trial, which employed adaptive randomization under a Bayesian hierarchical model, which entails using accumulating trial data to assign more patients to more effective treatments (38). A treatment was declared efficacious if the posterior probability of achieving >30% disease control rate (DCR) was >0.8, but a frequentist statistical analysis for overall survival was implemented.

### 6.2. Borrowing information in an umbrella trial

When a common treatment is investigated in all (or some) of the subtrials under an umbrella design, it may often be unclear whether the level of clinical activity of this treatment will be the similar in certain subgroups. When there is an expectation of similar clinical activity, investigators may choose to combine the results of corresponding subtrials receiving the same treatment arms (known as “complete pooling”). Alternatively, they may use statistical modeling approaches to “borrow information” from complementary subgroups when estimating the treatment effect in a particular subgroup. Here, we discuss considerations around when and how borrowing of information may be useful in an umbrella trial.

Firstly, if pooling or borrowing of information is to be employed, it must be carefully justified (58). For instance, the rationale for borrowing in basket trials is the shared drug target or disease symptom, such as recurrent fever flares in the case of the CLUSTER trial (68). In umbrella trials, a common control may be justified in some instances, say if the mechanism of action of the control is not dependent on the disease subtype, and the disease subtypes do not differ in prognosis; this would provide a logical basis for borrowing of information.

However, unlike the basket trial setting where pooling or borrowing of information is common, borrowing of information in umbrella trials is often viewed as an unfavorable approach because of the different hypotheses tested in the subtrials (4). Only six of 38 trials (15.8%) in our review did not perform standalone analyses of subtrials. Several umbrella trials included in our review are still ongoing, though, and this may consequently be an underestimate with pooling methods possibly planned for future analyses. Of the six studies, the use of simple approaches (i.e., combining all data from similar treatments) was predominant (4/6, 66.7%), while two trials (38, 39) used Bayesian methods.

Another possible reason for the low use of borrowing of information techniques in umbrella trials to date, is that our review suggests there is currently limited methodology around borrowing techniques tailored to the umbrella context. Only a few exceptions to this statement exist (4, 48–50). In particular, Kang et al. (49) recently proposed a hierarchical Bayesian clustering of multiple biomarker subgroups (HCOMBS) methodology. This approach allows clustering at interim and final analyses for a non-randomized phase II umbrella trial. HCOMBS uses a hierarchical Bayesian model to enable borrowing of information at the final analysis, with treatment arms clustered into low, moderate and high effect subgroups. Borrowing then takes place only within these three subgroups. One could argue that given treatments showing inactivity are dropped at interim analysis in Kang et al. (49), it is more likely that a homogeneous set of treatment effects will be observed at the final analysis and borrowing within subgroups makes more sense.

Yee et al. (4) considers the biomarker-strategy (also called marker-stratified) design (69) as a special case of an umbrella design where pooling is performed. We note that the biomarker-strategy design does not strictly fall under the auspices and aims of an umbrella trial design, although treatments are evaluated in a single disease setting. This is because the focus is not to assign all eligible marker positive patients to targeted experimental treatments under investigation, which is the basic principle for an umbrella design. The SUSKES trial (23) framework (Figure 2–top panel) is arguably similar to a biomarker-strategy design, except that biomarker screening is the basis of assignment to the “strategy” and “non-strategy” arms, and not randomization as is the case in the biomarker-strategy design. The rationale for complete pooling here makes sense because the interest is in whether the biomarker is prognostic. However, there is no justification for partial borrowing of information in this context. We note though that the strategy arm of some variants of the biomarker-strategy design (e.g., when multiple biomarkers are involved), will indeed match the premise of an umbrella design. Borrowing of information within the strategy arm, across a common control, may then be reasonable.

Zang et al. (57) proposed a Bayesian adaptive design to enable information sharing for the control arm only. Their approach captures the similarity of response rates in the control arm across the different biomarker subgroups using a Bayesian hierarchical model. However, the authors noted that certain assumptions warrant consideration; for example, the mechanism of action of the common drug or control should be similar in the different subgroups. We note here then that the choice of control arm(s) should always be made based on what is most appropriate for each subtrial; one should not seek to use a common control to gain the potential advantages of borrowing of information if alternative distinct control treatments would be more natural comparators.

The efficiencies realized by information borrowing—such as reduced sample size, higher power, and reduced type I error—have previously been demonstrated [see, e.g., Berry et al. (8)]. However, we caution against an expectation that information borrowing will always be beneficial. One major challenge to implementing information borrowing is the “mixed null” scenario—a case where a clinically meaningful effect is observed only in some subgroups, while in other subgroups the treatment is ineffective. In this case, information borrowing can lead to a higher false positive rate. For example, the approach in Kang et al. (49) showed desirable control of the overall family-wise error rate (FWER), provided effective arms had large effect sizes.

We also note that one fundamental question when borrowing information across subtrials is the amount of information to be borrowed and the robustness of the proposed approach to different treatment effect configurations. In this regard, the use of an analysis strategy that facilitates borrowing of information may only be most effective with extensive preliminary work to investigate the exact approach taken. One special sub-case in relation to the choice of borrowing technique is whether to use complete pooling vs. a more complex hierarchical method. Hierarchical techniques have been demonstrated to be superior in many settings (8, 16). This is unsurprising since complete pooling ignores the potential heterogeneity in the treatment effects that may be present across the subgroups. In this way, if information is to be borrowed, we believe the decision to be made should relate to the Bayesian approach adopted, with complete pooling rarely used.

Finally, we highlight that Bayesian approaches to borrowing of information need not be complex. For example, in the case of two subtrials, a hierarchical approach that requires estimation of the variability across arms may not be practical. Hence, one could use expert clinical opinion as weights to adjust the borrowing [see, e.g., Turner et al. (70)]. The advantage of such an approach is that uncertainty about similarity of parameters (i.e., mean response across arms or treatment effects) directly corresponds to the weights for each subtrial; this can be more intuitive to communicate.

## 7. Related master protocol designs

The umbrella design shares several principles with other master protocol designs—basket and platform trials—as their basic frameworks can be used or extended to answer multiple treatment related questions (i.e., studying multiple therapies, multiple diseases, or both). As such, a class of complex/hybrid master protocols whose designs leverage the efficiencies of umbrella and platform or basket trials under a single protocol have emerged. Our review identified several such trials, including SHIVA, NCI-MATCH, I-SPY 2, MyPathway, Paediatric-MATCH, NCI-MPACT, CUSTOM, CREATE, TAPUR, and SAFIR. For example, the NCI-MATCH trial has both umbrella and basket features; patients with at least 15 different cancers are enrolled into seven sub-protocols defined by targeted treatment-linked biomarkers (71). Some authors have described NCI-MATCH as an umbrella trial (3, 21), some have labeled it as a basket trial (1, 21), while others (3, 7) agree it fits neither classification.

The trial infrastructure of these hybrid designs is beneficial as more questions can be answered in a single trial compared to a standard umbrella, basket or platform trial. Notably, this can provide a convenient approach to understanding the potential heterogeneity of response across biomarker and targeted-therapy pairs. With advances in personalized medicine, umbrella designs may evolve to be more complex to accommodate more nuanced assignment of patients; that is, treatment assignment based on multiple characteristics (e.g., biomarkers), mechanisms of disease drug activity, disease pathways or clinical symptoms that the treatment targets. We caution that these designs will demand greater logistical and statistical considerations to ensure optimal efficiency; greater complexity does not guarantee more efficiency.

## 8. What don't we know? Open questions for umbrella trials

The design of umbrella trials has largely been motivated by drug development in oncology settings. The use of binary endpoints (practical for detection of early response), single-arm setup, small sample sizes, and well-defined and validated biomarkers is commonplace. Umbrella trials in non-oncology settings raise several open questions that necessitate methodological research. First, continuous, longitudinal, and even composite outcomes are more common in non-cancer trials. The current methodological landscape for umbrella trials does not well accommodate such outcome measures. That is, there is need for relevant methodology to enable application of umbrella designs in settings with these non-binary outcomes. In a basket trial setting, Chu and Yuan (12) proposed a Bayesian latent subgroup (BLAST) analysis that jointly modeled treatment response and longitudinal biomarker measurements. This may help motivate extensions to the umbrella setting.

Secondly, biomarkers targeted in non-oncology trials may be unreliable, partly attributable to the fact that the use of biomarkers in targeted therapy discoveries is still nascent in several disease areas, unlike in cancer research. Adaptive randomization could be useful to counter the higher likelihood of biomarker-treatment mismatch. Another biomarker-use related consideration is the dichotomization of continuous biomarkers, an approach that is well-known to lead to loss of information (72). Currently, there is no standard approach to defining umbrella trial subgroups on the basis of continuous markers. Some authors (73) have shown the potential of machine learning methods to stratify quantitative prognostic biomarkers, but more work is needed on continuous predictive biomarkers. The inclusion of the “non-match” arm further introduces some challenges. Specifically, the composition of this patient population can shift over time if new targeted treatments are added or dropped from the trial. Subsequently, results from this subtrial belong to a poorly defined patient population (i.e., unbiased sample of the biomarker negative population) for drug labeling purposes and are hard to interpret.

Thirdly, when there are many biomarkers (and linked treatments) to be evaluated, the level of efficiency of the umbrella design is debatable. The adaptive signature design, where several candidate biomarkers can be combined to formulate biomarker defined subgroups (74), potentially has utility or can be integrated with the umbrella design. The potential use and advantages of adaptive signature designs have been reported elsewhere (19, 74) and may be helpful given the immense logistical considerations that are necessary when investigating several biomarker-treatment pairs (4).

Fourthly, better approaches to performing sample size calculations remains an area of interest for several variants of the umbrella design. For instance, the rarity of subgroups limits the feasibility of randomization and calls for better approaches to keep the sample size low in randomized umbrella designs. Nearly all umbrella trials in our review adopted a simplistic approach to sample size calculation—performing sample size calculation independently for each subtrial. A pertinent question is whether this approach is (overly) conservative. The use of Bayesian methods that enable borrowing of internal (i.e., across subgroups) or external (i.e., historical controls) information are promising. Key to the development of relevant methodology for “optimal” (or more generally efficient) umbrella trial design, may be the specification of appropriate operating characteristics criteria to control during design.

Lastly, we envision that widespread implementation of umbrella trials in non-oncology settings will further stimulate methodological research on novel methods for their design and analysis. In particular, our review established the use of Bayesian methods is currently limited. Given that the concept of information borrowing is indeed plausible in some umbrella designs, the best strategies to undertake this, and in which cases borrowing may provide efficiency advantages must be explored further. The extensive work on Bayesian methodologies for basket trials provides an effective starting point for investigation in the umbrella setting.

## 9. Discussion

This review assesses the current landscape of methodology for umbrella trials, exploring current practice in published and ongoing studies, alongside the state-of-the-art in statistical methods. In particular, Section 8 brings about commentary with a non-oncology focus, to call for further research in umbrella trials for other disease areas. In this way, our work distinguishes itself from previous reviews (2, 5–7, 21).

Like many recent master protocol reviews (2, 5–7), we observed that the use of umbrella designs is still predominantly in oncology settings, but this landscape may be set to change in the next decade as precision medicine gains foothold in other disease areas. Furthermore, we established that most umbrella trials have been designed and implemented as a series of independent subtrials. This is unsurprising because the development of more complex statistical methods has not been as vibrant as in the basket trial setting. One key consideration leading to the current practice is that the evaluation of different targeted therapies in umbrella trials may imply that no borrowing of information can take place. However, previous work for example by Kang et al. (49) demonstrates this not to be the case.

Our review uncovers several areas for future methodological development. In particular, there is great room for the use of Bayesian methods in umbrella trials to increase. One specific area of application is the idea of borrowing of information for certain umbrella designs. The advantages of borrowing of information, such as increased power, imply that there may be a reduction in required sample size (8), but this requires further exploration. However, researchers should equally be aware of the limitations of borrowing information; when a drug works in some subgroups and not others, borrowing can inflate the type I error, and if a drugs works only in one subgroup, borrowing can lead to reduced power in that specific subgroup (8, 75). Therefore existing methods and proposals need to be evaluated carefully prior to their application for the primary analysis of a trial. Sample size determination in umbrella trials is an area of limited work currently and methods to consider this as a joint problem, rather than a subtrial specific problem, may also be impactful. Treating this as a joint problem especially when allowing for the use of adaptive designs, opens up the possibility for many flexible approaches to the design of an umbrella trial. Zheng et al. (76) offer a solution to Bayesian basket trials, of which the sample size is determined accounting for various degrees of borrowing pairwisely. This proposal could be highly relevant to the umbrella designs visualized in Figures 1C,D, when borrowing is enabled for the same treatment across subtrials Best practice guidance on how to employ particular broad classes of design will thus be required.

In addition to expanding the utility of umbrella designs in non-oncology settings, their successful uptake and implementation will, in our opinion, largely depend on two key factors. First, there is a need to improve the reporting of ongoing umbrella trials. We envision this as an important step toward increasing uptake of innovative designs. The dearth of information regarding, e.g., sample size computation, analysis strategies, and control of error rates, make learning lessons for future implementations challenging. Relatedly, fundamental aspects such as the purposes and objectives with an umbrella trial should be clearly stated to enhance further understanding of the design. Secondly, the improved efficiency of umbrella trials depends on development of efficient statistical methods. Our review points to several directions of methodology development, as noted above. These considerations may be compounded further by the increased utilization of more complex designs with umbrella and basket trial features.

We further draw attention to some recent regulatory position on complex clinical trials (including umbrella trials), that “*the rationale for the complexity of the design and conduct of a complex clinical trial needs to be explained in clear terms and justified in the protocol(s) and related documentation*” (77). Besides, the regulators address the need for a “scientific rationale” or “overall hypothesis” that defines the “*scientifically sound relationship(s) between the research questions of the sub-protocols*”. The scientific rationale here is different from the statistical hypothesis that is tested within a s specific sub-protocol or subtrial.

We acknowledge some limitations to this review. First, our statistics regarding the characteristics of identified umbrella trials are impacted by the lack of published protocol and/or final reports for many of the identified trials. While we sought to offset this through an extensive data extraction procedure—e.g., seeking relevant information from clinicaltrials.gov as well as published articles—it may be the case that they under-/over-imply the use of certain techniques. It is part of our plans to update this review in the near future to include recent non-oncology umbrella trials, for instance in emerging areas such as COVID-19. Secondly, the systematic search was only performed in a single electronic database, PubMED. However, this limitation was offset by the fact that results from the PubMED search were complemented with those from four very recent systematic reviews (5–7, 21) undertaken/published in the period this review was ongoing (between 2018 and 2021). The four aforementioned systematic reviews involved searches in MEDLINE, EMBASE, CENTRAL, and trial registries (clinicaltrials.gov and ISRCTN). Thirdly, lack of standardized nomenclatures and trial misclassification of master protocols (21) may have limited the identification of some studies, especially outside of oncology where these terminologies are perhaps not widespread. However, our search strategy was robust and we included trials based on them meeting the umbrella definition rather than solely on how they are labeled by authors or have been classified by previous reviews.pre-specified prioritization approach In addition, we focused on the design and analysis of umbrella trials. But, the successful conduct of umbrella trials also depends on practical considerations including proper data infrastructures to handle large-scale genomic data, and informatics systems to process data and determine treatment eligibility/assignment (4). Even then, the ethical challenges posed by umbrella trials need careful consideration (78). Furthermore, the use of the estimand framework in master protocols has become an area of growing consideration (79, 80). This may lead to substantial transformations in the way umbrella trials should be analyzed and reported over the coming years which are not discussed in our work. A final note is that despite the non-oncology focus in several areas of this article, this does not justify umbrella trials as well-established in oncology with no areas of improvement. In principle, many suggestions provided herein may also be applied to improving umbrella trials also in oncology.

To conclude, we hope that this review will help provide an in-depth understanding of the design and analysis considerations for umbrella trials for statisticians and non-statisticians alike, especially those working in non-oncology settings, and that it will help motivate much future research. The potential for umbrella designs to assist with expedited drug development across therapeutic areas is vast; in our opinion, we are not far from seeing this become reality.

## Data availability statement

The original contributions presented in the study are included in the article/Supplementary material, further inquiries can be directed to the corresponding author/s.

## Author contributions

JW, MG, HZ, and LO designed the study. LO analyzed the review data. All authors contributed to the literature review and drafted the manuscript. All authors contributed to the article and approved the submitted version.

## Funding

HZ was funded by a CRUK Research Fellowship (RCCPDF\100008). JW was funded by a NIHR Research Professorship (NIHR301614).

## Conflict of interest

The authors declare that the research was conducted in the absence of any commercial or financial relationships that could be construed as a potential conflict of interest.

## Publisher's note

All claims expressed in this article are solely those of the authors and do not necessarily represent those of their affiliated organizations, or those of the publisher, the editors and the reviewers. Any product that may be evaluated in this article, or claim that may be made by its manufacturer, is not guaranteed or endorsed by the publisher.
