# Supplementary material for: Design and analysis of umbrella trials: Where do we stand?
Source: Front Med (Lausanne). 2022 Oct 12;9:1037439. doi: 10.3389/fmed.2022.1037439 (PMC9596938; doi:10.3389/fmed.2022.1037439)
Supplement: Supplementary file 1 [file Data_Sheet_1.PDF]

Supplementary Material

1 LITERATURE SEARCH STRATEGY

The following literature search term was used in PubMed:

(“basket design”) OR (“basket study”) OR (”basket trial”) OR (“matrix approach”) OR (“matrix design”) OR (“matrix study”) OR (“matrix trial”) OR (“platform approach”) OR (“platform design”) OR (“platform study”) OR (“platform trial”) OR (“umbrella approach”) OR (”umbrella design”) OR (“umbrella study”) OR (“umbrella trial”) OR (“master protocol”)

These search terms were selected based on assessment of key master protocol publications and previously published reviews of master protocols. We initially considered additional use of the word bucket, but this was found to produce no articles in PubMed when followed by the word design, study, trial, or approach. The words basket and platform were retained despite our focus on umbrella trials because of previous misclassifications of master protocol designs.

The initial search was performed on 16th October 2019. Owing to the length of time it took to assess the returned articles for relevance, a second search was performed to update the list of returned articles on 12th May 2021. The initial search returned 1413 articles for review; the updated search returned an additional 376 articles. Two reviewers were assigned to each article to assess it for relevance to umbrella trials. Identified relevant papers that did not relate to a conducted umbrella trial were noted for subsequent synthesis into the narrative component of our review. For identified umbrella trials, data was extracted using a data extraction template described further below.

2 DATA EXTRACTION TEMPLATE

Data was extracted on the elements listed in Table S1 for all identified umbrella trials.

Table S1. Data extracted from umbrella trials included in the systematic review

- If there's a Study ID (e.g., from *clinicaltrials.gov*), what is it?
- If there's a trial acronym, what is it?
- Articles it is mentioned in?
- Additional sources of information?
- What phase of trial is it (e.g., phase II)?
- What disease area is it (e.g., oncology)?
- If oncology, what cancer type (e.g., breast, lung)?
- How many modules are there in the design?
- Are the modules mutually exclusive or overlapping?
- Are patients eligible for multiple modules?
- What is the planned sample size per module?
- What is the actual sample size per module?
- What is/are the primary outcome(s)?
- Is the primary outcome the same in each module?
- Is the effect size the same or different in each module?
- Did it use a randomised, single-arm, or mixed design?
- If it is a randomised design, is the control arm the same across modules?
- Is it an adaptive or non-adaptive design?
- Is there a method given for how the trial was designed (e.g., a cited methods paper or a given formula/description)?
- Did the sample size calculation use a separate or pooled approach?
- What is the statistical analysis method for the primary outcome?
- Is the analysis Bayesian or frequentist?
- Is the analysis pooled or separate?
- If pooled, what is the pooling method?
- What error-rates did they control?
- Is the error-rate control consistent with design?
- Is there missing data?
- What is the method to deal with missing data?
- How to deal with patients eligible for multiple treatments/subgroups

3 PRISMA FLOWCHART

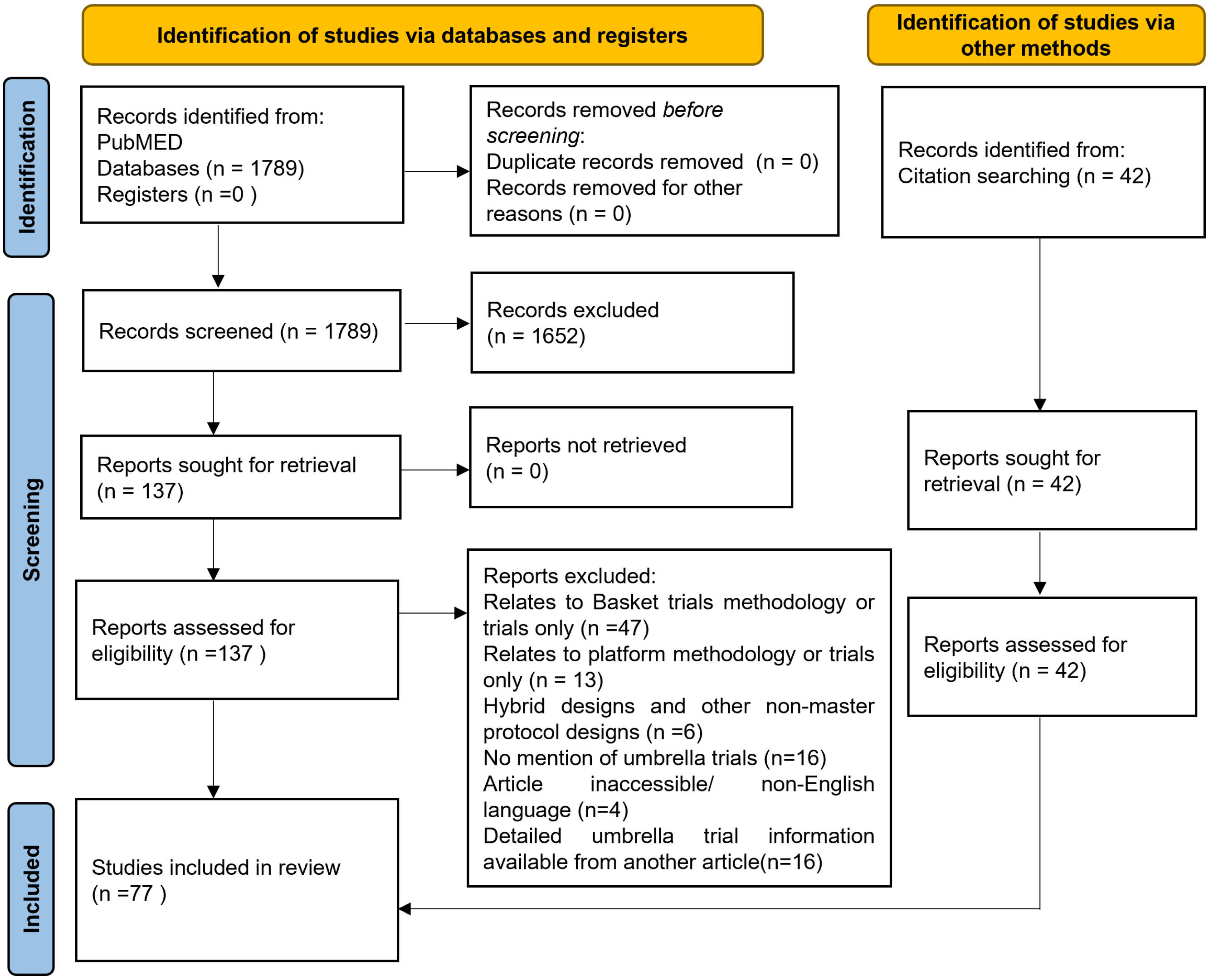

Figure S1. The PRISMA flow-diagram of articles selected and included in the systematic review.

4 LIST OF UMBRELLA TRIALS INCLUDED IN THE SYSTEMATIC REVIEW

Table S2: List of Umbrella trials included in the systematic review

ID	Acronym	Trial phase	Disease setting	References
NCT01641367	ACTG A5288	IV	Non-oncology	(1, 2)
NCT02194738, NCT02193282, NCT02201992, NCT02595944	ALCHEMIST	III	Oncology	(3)
NCT03994796	Alliance	II	Oncology	(4)
NCT03699449	AMBITION	II	Oncology	(5)
ISRCTN25859465	ATLANTIS	II	Oncology	(6)
NCT00409968, NCT00411632, NCT00411671, NCT00410189, NCT00410059	BATTLE-1	II	Oncology	(7, 8)
NCT01248247	BATTLE-2	II	Oncology	(9, 8)
NCT02546661	BISCAY	I	Oncology	(10)
NCT02574078	CheckMate 370	I/II	Oncology	(11)
NCT02314481	DARWINII	II	Oncology	(12)
NCT01760005	DIAN-TU	II/III	Non-oncology	(13)
NCT03267589	ENGOT-OV30/ NSGO	II	Oncology	(14)
ISRCTN90061546	FOCUS4	II/III	Oncology	(15, 16)
NCT03805399	FUTURE	I/II	Oncology	(17)
NCT03334617	HUDSON	II	Oncology	(14)
ACTRN12612000777897	IMPaCT	II	Oncology	(18)
NCT03851445, NCT02154490, NCT02766335, NCT02785913, NCT02785939, NCT02965378, NCT02926638, NCT03373760, NCT03377556, NCT02785952	Lung-MAP	II/III	Oncology	(19, 20, 21, 22)
UMIN000027552	MASTER KEY	Unclear	Oncology	(23, 24)
NCT02551055	MLN1117	I	Oncology	(14)
NCT02291289	MODUL	II	Oncology	(25, 26)
NCT03424005, NCT03280563, NCT03555149, NCT03193190, NCT03281369, NCT03337698	MORPHEUS	I/II	Oncology	(27, 14)
NCT03732703	MyDRUG	I/II	Oncology	(28)
NCT03158389, NCT00903734, NCT00895128, NCT00895362, NCT00895687	N2M2	I/II	Oncology	(29)
NCT02664935	NLMT	II	Oncology	(12, 30, 31, 32, 33)
ISRCTN16426935	OCTOPUS (AZD2014)	II	Oncology	(34)
NCT02213289	PANGEA	II	Oncology	(35)
NCT03182634	plasmaMATCH	II	Oncology	(36)

Table S2 (continued..): List of Umbrella trials included in the systematic review

ID	Acronym	Trial phase	Disease setting	References
ISRCTN14879538, ISRCTN75002153, NCT04151277	PRIMUS (Precision Panc)	Unclear	Oncology	(37, 38)
NCT02688894	SUKSES	II	Oncology	(39)
NCT01941940, NCT01941095, NCT01951170, NCT01987479, NCT01988012, NCT01995201, NCT02001987, NCT02011334, NCT02031471, NCT02046603, NCT02046616	TOZURA	III/IV	Non-oncology	(40, 41, 42, 43)
NCT03292250, NCT03356587	TRIUMPH	II	Oncology	(14, 44)
NCT03574402	TRUMP	II	Oncology	(14)
NCT01940497, NCT01926886, NCT02194166, NCT01964391, NCT02040935	UmbHER1	III	Oncology	(45)
NCT03088059	UPSTREAM	II	Oncology	(46)
NCT02299648	VIKTORY	II	Oncology	(47)
NCT01781338	WSG ADAPT	II/III	Oncology	(14, 48)
NCT00903734, NCT00895128, NCT00895362, NCT00895687		I	Oncology	(14)
NCT02951091	K-Umbrella	II	Oncology	(49)

5 PICOS CRITERIA AND PRISMA CHECKLIST

This review was conducted in accordance with the Preferred Reporting Items for Systematic Reviews and Meta-Analysis (PRISMA) guidance(50).

Table S3. PICOS (population, intervention, comparator, outcomes, study design) criteria for the systematic review

Category	Inclusion criteria
Population	Humans
Intervention	No restrictions
Comparator	No restrictions
Outcomes	No restrictions
Study design	Umbrella trial*
Other	English language publications and conference abstracts with either statistical methodology, trial results or published protocols.

*__Note__ – The following working definitions were adopted:

- Basket trial: Evaluates one biomarker-targeted therapy on multiple diseases or multiple disease subtypes;
- Umbrella trial: Evaluates multiple targeted therapies for different subgroups of a disease.
- Platform trial: Evaluates multiple experimental treatments in a single disease in a perpetual manner.

Table S4: PRISMA checklist

Section and Topic	Item #	Checklist item	Location where item is reported
**TITLE**			
Title	1	Identify the report as a systematic review.	1
**ABSTRACT**			
Abstract	2	See the PRISMA 2020 for Abstracts checklist.	1
**INTRODUCTION**			
Rationale	3	Describe the rationale for the review in the context of existing knowledge.	2
Objectives	4	Provide an explicit statement of the objective(s) or question(s) the review addresses.	2
**METHODS**			
Eligibility criteria	5	Specify the inclusion and exclusion criteria for the review and how studies were grouped for the syntheses.	3
Information sources	6	Specify all databases, registers, websites, organisations, reference lists and other sources searched or consulted to identify studies. Specify the date when each source was last searched or consulted.	3
Search strategy	7	Present the full search strategies for all databases, registers and websites, including any filters and limits used.	Supplementary material p1
Selection process	8	Specify the methods used to decide whether a study met the inclusion criteria of the review, including how many reviewers screened each record and each report retrieved, whether they worked independently, and if applicable, details of automation tools used in the process.	3
Data collection process	9	Specify the methods used to collect data from reports, including how many reviewers collected data from each report, whether they worked independently, any processes for obtaining or confirming data from study investigators, and if applicable, details of automation tools used in the process.	3, Table S1
Data items	10a	List and define all outcomes for which data were sought. Specify whether all results that were compatible with each outcome domain in each study were sought (e.g. for all measures, time points, analyses), and if not, the methods used to decide which results to collect.	NA
	10b	List and define all other variables for which data were sought (e.g. participant and intervention characteristics, funding sources). Describe any assumptions made about any missing or unclear information.	Table S1

Table S4 (continued..): PRISMA checklist

Section and Topic	Item #	Checklist item	Location where item is reported
Study risk of bias assessment	11	Specify the methods used to assess risk of bias in the included studies, including details of the tool(s) used, how many reviewers assessed each study and whether they worked independently, and if applicable, details of automation tools used in the process.	NA
Effect measures	12	Specify for each outcome the effect measure(s) (e.g. risk ratio, mean difference) used in the synthesis or presentation of results.	NA
Synthesis methods	13a	Describe the processes used to decide which studies were eligible for each synthesis (e.g. tabulating the study intervention characteristics and comparing against the planned groups for each synthesis (item #5)).	NA
	13b	Describe any methods required to prepare the data for presentation or synthesis, such as handling of missing summary statistics, or data conversions.	3
	13c	Describe any methods used to tabulate or visually display results of individual studies and syntheses.	3
	13d	Describe any methods used to synthesize results and provide a rationale for the choice(s). If meta-analysis was performed, describe the model(s), method(s) to identify the presence and extent of statistical heterogeneity, and software package(s) used.	3
	13e	Describe any methods used to explore possible causes of heterogeneity among study results (e.g. subgroup analysis, meta-regression).	NA
	13f	Describe any sensitivity analyses conducted to assess robustness of the synthesised results.	NA
Reporting bias assessment	14	Describe any methods used to assess risk of bias due to missing results in a synthesis (arising from reporting biases).	NA
Certainty assessment	15	Describe any methods used to assess certainty (or confidence) in the body of evidence for an outcome.	NA
**RESULTS**			
Study selection	16a	Describe the results of the search and selection process, from the number of records identified in the search to the number of studies included in the review, ideally using a flow diagram.	4, Figure S1
	16b	Cite studies that might appear to meet the inclusion criteria, but which were excluded, and explain why they were excluded.	3
Study characteristics	17	Cite each included study and present its characteristics.	4 – 12, Table S2

Table S4 (continued..): PRISMA checklist

Section and Topic	Item #	Checklist item	Location where item is reported
Risk of bias in studies	18	Present assessments of risk of bias for each included study.	NA
Results of individual studies	19	For all outcomes, present, for each study: (a) summary statistics for each group (where appropriate) and (b) an effect estimate and its precision (e.g. confidence/credible interval), ideally using structured tables or plots.	NA
Results of syntheses	20a	For each synthesis, briefly summarise the characteristics and risk of bias among contributing studies.	NA
	20b	Present results of all statistical syntheses conducted. If meta-analysis was done, present for each the summary estimate and its precision (e.g. confidence/credible interval) and measures of statistical heterogeneity. If comparing groups, describe the direction of the effect.	NA
	20c	Present results of all investigations of possible causes of heterogeneity among study results.	NA
	20d	Present results of all sensitivity analyses conducted to assess the robustness of the synthesised results.	NA
Reporting biases	21	Present assessments of risk of bias due to missing results (arising from reporting biases) for each synthesis assessed.	NA
Certainty of evidence	22	Present assessments of certainty (or confidence) in the body of evidence for each outcome assessed.	NA
**DISCUSSION**			
Discussion	23a	Provide a general interpretation of the results in the context of other evidence.	14 – 15
	23b	Discuss any limitations of the evidence included in the review.	15
	23c	Discuss any limitations of the review processes used.	15
	23d	Discuss implications of the results for practice, policy, and future research.	15
**OTHER INFORMATION**			
Registration and protocol	24a	Provide registration information for the review, including register name and registration number, or state that the review was not registered.	3
	24b	Indicate where the review protocol can be accessed, or state that a protocol was not prepared.	NA
	24c	Describe and explain any amendments to information provided at registration or in the protocol.	NA
Support	25	Describe sources of financial or non-financial support for the review, and the role of the funders or sponsors in the review.	16

Table S4 (continued..): PRISMA checklist

Section and Topic	Item #	Checklist item	Location where item is reported
Competing interests	26	Declare any competing interests of review authors.	15
Availability of data, code and other materials	27	Report which of the following are publicly available and where they can be found: template data collection forms; data extracted from included studies; data used for all analyses; analytic code; any other materials used in the review.	16, supplementary material p2

6 PRISMA CHECKLIST FOR ABSTRACTS

Table S5. PRISMA checklist for Abstracts

Section and Topic	Item #	Checklist item	Reported (Yes/No)
**TITLE**			
Title	1	Identify the report as a systematic review.	Y
**BACKGROUND**			
Objectives	2	Provide an explicit statement of the main objective(s) or question(s) the review addresses.	Y
**METHODS**			
Eligibility criteria	3	Specify the inclusion and exclusion criteria for the review.	Y
Information sources	4	Specify the information sources (e.g. databases, registers) used to identify studies and the date when each was last searched.	Y
Risk of bias	5	Specify the methods used to assess risk of bias in the included studies.	N
Synthesis of results	6	Specify the methods used to present and synthesise results.	Y
**RESULTS**			
Included studies	7	Give the total number of included studies and participants and summarise relevant characteristics of studies.	Y
Synthesis of results	8	Present results for main outcomes, preferably indicating the number of included studies and participants for each. If meta-analysis was done, report the summary estimate and confidence/credible interval. If comparing groups, indicate the direction of the effect (i.e. which group is favoured).	Y
**DISCUSSION**			
Limitations of evidence	9	Provide a brief summary of the limitations of the evidence included in the review (e.g. study risk of bias, inconsistency and imprecision).	Y
Interpretation	10	Provide a general interpretation of the results and important implications.	Y
**OTHER**			
Funding	11	Specify the primary source of funding for the review.	N
Registration	12	Provide the register name and registration number.	N

REFERENCES

1 .Grinsztejn B, Hughes M, Ritz J, Salata R, Mugyenyi P, Hogg E, et al. Third-line antiretroviral therapy in low-income and middle-income countries (ACTG A5288): A prospective strategy study. *The Lancet HIV* **6** (2019) e588–600.

2 .Park J, Ford N, Xavier D, Ashorn P, Grais R, Bhutta Z, et al. Randomised trials at the level of the individual. *Lancet Global Health* **9** (2021) E691–700.

3 .Gerber D, Oxnard G, Govindan R. ALCHEMIST: Bringing genomic discovery and targeted therapies to early-stage lung cancer. *Clinical Pharmacology & Therapeutics* **97** (2015) 447–50.

4 .Kang D, Coffey C, Smith B, Yuan Y, Shi Q, Yin J. Hierarchical Bayesian clustering design of multiple biomarker subgroups (HCOMBS). *Statistics in Medicine* **40** (2021) 2893–921.

5 .Lee J, Yi J, Kim H, Lim J, Kim S, Nam B, et al. An umbrella study of biomarker-driven targeted therapy in patients with platinum-resistant recurrent ovarian cancer: A Korean Gynecologic Oncology Group study (KGOG 3045), AMBITION. *Japanese Journal of Clinical Oncology* **49** (2019) 789–92.

6 .Fulton B, Jones R, Powles T, Crabb S, Paul J, Birtle A, et al. ATLANTIS: A randomised multi-arm phase II biomarker-directed umbrella screening trial of maintenance targeted therapy after chemotherapy in patients with advanced or metastatic urothelial cancer. *Trials* **21** (2020) 344.

7 .Kim E, Herbst R, Wistuba I, Lee J, Blumenschein G, Tsao A, et al. The BATTLE trial: Personalizing Therapy for Lung Cancer. *Cancer Discovery* **1** (2011) 44–53.

8 .Liu S, Lee J. An overview of the design and conduct of the BATTLE trials. *Chinese Clinical Oncology* **4** (2015) 1–13.

9 .Papadimitrakopoulou V, Lee J, Wistuba I, Tsao A, Fossella F, Kalhor N, et al. The BATTLE-2 Study: A biomarker-integrated targeted therapy study in previously treated patients with advanced non-small-cell lung cancer. *J Clin Oncol* **34** (2016) 3638–47.

10 .Powles T, Carroll D, Chowdhury S, Gravis G, Joly F, Carles J, et al. An adaptive, biomarker-directed platform study of durvalumab in combination with targeted therapies in advanced urothelial cancer. *Nature Medicine* **27** (2021) 793–801.

11 .Spigel D, Reynolds C, Waterhouse D, Garon E, Chandler J, Babu S, et al. Phase 1/2 study of the safety and tolerability of nivolumab plus crizotinib for the first-line treatment of anaplastic lymphoma kinase translocation — Positive advanced non–small cell lung cancer (CheckMate 370). *Journal of Thoracic Oncology* **13** (2018) 682–8.

12 .Hiley C, Quesne JL, Santis G, Sharpe R, Gonzalez de Castro D, Middleton G, et al. Challenges in molecular testing in non-small-cell lung cancer patients with advanced disease. *Lancet* **388** (2016) 1002–11.

13 .Bateman R, Benzinger T, Berry S, Clifford D, Duggan C, Fagan A, et al. The DIAN-TU next generation Alzheimer's prevention trial: Adaptive design and disease progression model. *Alzheimer's & Dementia* **13** (2017) 8–19.

14 .Janiaud P, Serghiou S, Ioannidis J. New clinical trial designs in the era of precision medicine: An overview of definitions, strengths, weaknesses, and current use in oncology. *Cancer Treatment Reviews* **73** (2019) 20–30.

15 .Kaplan R, Maughan T, Crook A, Fisher D, Wilson R, Brown L, et al. Evaluating many treatments and biomarkers in oncology: A new design. *Journal of Clinical Oncology* **31** (2013) 4562–70.

16 .Adams R, Brown E, Brown L, Butler R, Falk S, Fisher D, et al. Inhibition of EGFR, HER2, and HER3 signalling in patients with colorectal cancer wild-type for BRAF, PIK3CA, KRAS, and NRAS (FOCUS4-D): A phase 2–3 randomised trial. *The Lancet Gastroenterology and Hepatology* **3** (2018) 162–71.

17 .Jiang Y, Liu Y, Xiao Y, Hu X, Jiang L, Zuo W, et al. Molecular subtyping and genomic profiling expand precision medicine in refractory metastatic triple-negative breast cancer: The FUTURE trial. *Cell Research* **31** (2021) 178–86.

18 .Cowley M, Chang D, Pajic M, Johns A, Waddell N, Grimmond S, et al. Understanding pancreatic cancer genomes. *Journal of Hepatobiliary Pancreatic Sciences* **20** (2013) 549–56.

19 .Ferrarotto R, Redman M, Gandara D, Herbst R, Papadimitrakopoulou V. Lung-MAP–Framework, overview, and design principles. *Chinese Clinical Oncology* **4** (2015) 36.

20 .Herbst R, Gandara D, FR FH, Redman M, LeBlanc M, Mack P, et al. Lung Master Protocol (Lung-MAP)—A biomarker-driven protocol for accelerating development of therapies for squamous cell lung cancer: SWOG S1400. *Clinical Cancer Research* **21** (2015) 1514–24.

21 .Edelman M, Redman M, Albain K, McGary E, Rafique N, Petro D, et al. SWOG S1400C (NCT02154490)-A phase II study of palbociclib for previously treated cell cycle gene alteration-positive patients with stage IV squamous cell lung cancer (Lung-MAP substudy). *Journal of Thoracic Oncology* **14** (2019) 1853–9.

22 .Wade J, Langer C, Redman M, Aggarwal C, Bradley J, Crawford J, et al. A phase II study of GDC-0032 (taselisib) for previously treated PI3K positive patients with stage IV squamous cell lung cancer (SqNSCLC): LUNG-MAP sub-study SWOG S1400B. *Journal of Clinical Oncology* **35** (2017) 9054.

23 .Okuma H, Yonemori K, Shimizu T, Goto Y, Honma Y, Morizane C, et al. MASTER KEY project: A basket/umbrella trial for rare cancers in Japan. *Journal of Clinical Oncology* **36** (2018) TPS2598.

24 .Okuma H, Yonemori K, Narita S, Sukigara T, Hirakawa A, Shimizu T, et al. MASTER KEY Project: Powering Clinical Development for Rare Cancers Through a Platform Trial. *Clinical Pharmacology & Therapeutics* **108** (2020) 596–605.

25 .Lam M, Loree J, Pereira A, Chun Y, Kopetz S. Accelerating therapeutic development through innovative trial design in colorectal cancer. *Current Treatment Options in Oncology* **19** (2018) 11.

26 .Schmoll H, Arnold D, de Gramont A, Ducreux M, Grothey A, O'Dwyer P, et al. MODUL–A multicenter randomized clinical trial of biomarker-driven maintenance therapy following first-line standard induction treatment of metastatic colorectal cancer: An adaptable signal-seeking approach. *Journal of Cancer Research and Clinical Oncology* **144** (2018) 1197–204.

27 .Chau I, Haag G, Rahma O, Macarulla T, McCune S, Yardley D, et al. MORPHEUS: A phase Ib/II umbrella study platform evaluating the safety and efficacy of multiple cancer immunotherapy (CIT)-based combinations in different tumour types. *Annals of Oncology* **29** (2018) viii439–440.

28 .Kumar S, Paiva B, Anderson K, Durie B, Landgren O, Moreau P, et al. International Myeloma Working Group consensus criteria for response and minimal residual disease assessment in multiple myeloma. *The Lancet Oncology* **17** (2016) e328–46.

29 .Wick W, Dettmer S, Berberich A, Kessler T, Karapanagiotou-Schenkel I, Wick A, et al. N2M2 (NOA-20) phase I/II trial of molecularly matched targeted therapies plus radiotherapy in patients with newly diagnosed non-MGMT hypermethylated glioblastoma. *Neuro-oncology* **21** (2019) 95–105.

30 .Middleton G, Crack L, Popat S, , Swanton C, Hollingsworth S, et al. The National Lung Matrix Trial: Translating the biology of stratification in advanced non-small-cell lung cancer. *Annals of Oncology* **26** (2015) 2464–9.

31 .Redman M, Allegra C. The master protocol concept. *Seminars in Oncology* **42** (2015) 724–30.

32 .Harrow S, Hanna G, Faivre-Finn C, McDonald F, Chalmers A. The challenges faced in developing novel drug radiation combinations in non-small cell lung cancer. *Clinical Oncology* **28** (2016) 720–5.

33 .Lindsay C, Shaw E, Blackhall F, Blyth K, Brenton J, Chaturvedi A, et al. Somatic cancer genetics in the UK: Real-world data from phase i of the Cancer Research UK Stratified Medicine Programme. *ESMO Open* **3** (2018) 5.

34 .Banerjee S, Lewsley L, Clamp A, Gabra H, Herbertson R, Green C, et al. OCTOPUS: A randomised, multi-centre phase II umbrella trial of weekly paclitaxel+/- novel agents in platinum-resistant ovarian cancer—Vistusertib (AZD2014). *Journal of Clinical Oncology* **35** (2017) TPS5609.

35 .Catenacci D. Next-generation clinical trials: Novel strategies to address the challenge of tumor molecular heterogeneity. *Molecular Oncology* **9** (2015) 967–96.

36 .Turner N, Kingston B, Kilburn L, Kernaghan S, Wardley A, Macpherson I, et al. Circulating tumour DNA analysis to direct therapy in advanced breast cancer (plasmaMATCH): A multicentre, multicohort, phase 2a, platform trial. *The Lancet Oncology* **21** (2020) 1296–308.

37 .Graham J, Valle J, Evans T, Grose D, Paul J, Stobo J, et al. PRIMUS-001: An adaptive phase II study of FOLFOX-A (FOLFOX and nab-paclitaxel) versus AG (nab-paclitaxel and gemcitabine) in patients with metastatic pancreatic cancer, with integrated biomarker evaluation (ISRCTN75002153) – Part of Precision-Panc. *Journal of Clinical Oncology* **36** (2018) TPS4158.

38 .Valle J, Evans T, Jeffry T, McKay C, Dixon-Hughes J, Paul J, et al. Precision-Panc Master Protocol: Personalizing treatment for pancreatic cancer ISRCTN14879538—Part of Precision-Panc United Kingdom. *Journal of Clinical Oncology* **37** (2019) TPS460.

39 .Park S, Shim J, Mortimer P, Smith S, Godin R, Hollingsworth S, et al. Biomarker-driven phase 2 umbrella trial study for patients with recurrent small cell lung cancer failing platinum-based chemotherapy. *Cancer* **126** (2020) 4002–12.

40 .Bazzichi L, Nacci F, Sinigaglia L, Bianchino L, Caporali R. Subcutaneous tocilizumab alone or with a csDMARD in rheumatoid arthritis patients: Subanalysis of Italian data from a multicenter phase IIIb/IV trial. *Clinical Rheumatology* **38** (2019) 841–9.

41 .Choy E, Caporali R, Xavier R, Fautrel B, Sanmarti R, Bao M, et al. Effects of concomitant glucocorticoids in TOZURA, a common-framework study programme of subcutaneous tocilizumab in rheumatoid arthritis. *Rheumatology* **58** (2019) 1056–64.

42 .Choy E, Caporali R, Xavier R, Fautrel B, Sanmarti R, Bao M, et al. Subcutaneous tocilizumab in rheumatoid arthritis: Findings from the common-framework phase 4 study programme TOZURA conducted in 22 countries. *Rheumatology* **57** (2018) 499–507.

43 .Choy E, Caporali R, Xavier R, Fautrel B, Sanmarti R, Bernasconi C, et al. Subcutaneous Tocilizumab as monotherapy or in combination with a csDMARDs in patients with rheumatoid arthritis – Interim analysis of a large phase IV international umbrella study, ”Tozura”. *Annals of the Rheumatic Diseases* **75** (2016) 509–10.

44 .Chang H, Lee Y, Ko Y, Cho J, Choi J, Park K, et al. Prognostic value of CD200R1 mRNA expression in head and neck squamous cell carcinoma. *Cancers* **12** (2020) 1777.

45 .Pivot X, Poole C, Martín M, Gligorov J, Barrios C, Vrdoljak E, et al. An open-label, multinational, multicentre, phase IIIB umbrella study of subcutaneous trastuzumab with or without chemotherapy or pertuzumab in patients with HER2-positive early or metastatic breast cancer (UmbHER1): Interim safety results from early breast cancer studies. *Annals of Oncology* **27** (2016) vi63.

46 .Galot R, Licitra L, Tourneau CL, Guigay J, Tinhofer I, Kong A, et al. EORTC 1559-HNCG: A pilot study of personalized biomarker-based treatment strategy or immunotherapy in patients with recurrent/metastatic (R/M) squamous cell carcinoma of the head and neck (SCCHN)-“UPSTREAM”. *Journal of Clinical Oncology* **36** (2018) TPS6095.

47 .Lee J, Kim S, Kim K, Lee H, Kozarewa I, Mortimer P, et al. Tumor genomic profiling guides patients with metastatic gastric cancer to targeted treatment: The VIKTORY umbrella trial. *Cancer Discovery* **9** (2019) 1388–405.

48 .Hofmann D, Nitz U, Gluz O, Kates R, Schinkoethe T, Staib P, et al. WSG ADAPT – Adjuvant dynamic marker-adjusted personalized therapy trial optimizing risk assessment and therapy response prediction in early breast cancer: Study protocol for a prospective, multi-center, controlled, non-blinded, randomized, investigator initiated phase II/III trial. *Trials* **14** (2013) 261.

49 .Rha SY, Lee Ck, Kim HS, Jung M, Kim H, Kyun BW, et al. The first report of K-Umbrella Gastric Cancer Study: An open label, multi-center, randomized, biomarker-integrated trial for second-line treatment of advanced gastric cancer (AGC). *https://doi.org/10.1200/JCO.2022.40.16_suppl.4001* **40** (2022) 4001–4001. doi:10.1200/JCO.2022.40.16{_}SUPPL.4001.

50 .Page M, McKenzie J, Bossuyt P, Boutron I, Hoffmann T, Mulrow C, et al. The PRISMA 2020 statement: An updated guideline for reporting systematic reviews. *BMJ* **372** (2021) 372.
